# The Effect of Glycine Betaine on Nitrogen and Polyamine Metabolisms, Expression of Glycoside-Related Biosynthetic Enzymes, and K/Na Balance of Stevia under Salt Stress

**DOI:** 10.3390/plants12081628

**Published:** 2023-04-12

**Authors:** Abazar Ghorbani, Vali Ollah Ghasemi-Omran, Moxian Chen

**Affiliations:** 1 National Key Laboratory of Green Pesticide, Key Laboratory of Green Pesticide and Agricultural Bioengineering, Ministry of Education, Center for R&D of Fine Chemicals of Guizhou University, Guiyang 550025, China; 2 Department of Agronomy, Genetics and Agricultural Biotechnology Institute of Tabarestan, Sari Agricultural Science and Natural Resources University, Sari 68984, Iran

**Keywords:** glycine betaine, NaCl stress, polyamine metabolism, nitrogen metabolism, steviol glycosides

## Abstract

The beneficial role of glycine betaine (GB) in the adaptation of plants to abiotic stresses is well known; therefore, the study of physiological and molecular responses induced by exogenous GB under NaCl stress can provide a suitable reference for the application of this compound to enhance the adaptation of plants to salinity. The present study was conducted under in vitro conditions to evaluate the effect of GB (25 and 50 mM) on the growth, physiological, and molecular traits of *Stevia rebaudiana* during NaCl toxicity (50 mM). The results showed that applying NaCl treatment increased Na accumulation, induced oxidative stress, and disrupted N metabolism and K/Na homeostasis, which, as a result, decreased the stevia plant’s growth and biomass. However, application of GB improved the adaptation of NaCl-stressed plants by improving N metabolism and modulating the metabolism of polyamines. By increasing the activity of antioxidant enzymes, GB diminished oxidative stress, protected the plasma membrane, and restored photosynthetic pigments under NaCl toxicity. By reducing Na accumulation and increasing K accumulation, GB maintained the K/Na balance and reduced the effects of toxicity caused by the high Na concentration in stevia leaves. GB increased the leaf accumulation of rebaudioside A in NaCl-stressed plants by modulating the expression of genes (*KAH*, *UGT74G1*, *UGT76G1*, and *UGT85C2*) involved in the sugar compounds of the stevia plants. Our results provide a broad understanding of GB-induced responses in NaCl-stressed plants, which can help increase our knowledge of the role of GB in the defense mechanisms of plants under abiotic stresses.

## 1. Introduction

Salt and drought stresses are not only a serious threat to the food security of people on earth but also affect the quality of medicinal plant products, which are recognized as the most important consequences of global warming [1,2]. Every year, nearly 2 million hectares of arable land are affected by salinity stress, which is associated with a drop in productivity in agricultural or medicinal plants [3]. High concentrations of toxic ions, mainly Na, oxidative stress, leaf senescence, and hyperosmotic stress are the most important destructive effects induced by salinity stress in plants [4,5]. The biochemical and molecular changes brought about by salinity, which raise internal concentrations of osmoprotectants and antioxidants or regulate the expression of ion transporters (NHXs or HKT1), can be a useful tactic to promote plant adaptation [6,7]. Nevertheless, due to the complexities and controversies associated with transgenic crops, this strategy has practically failed to completely solve the problems in this field [1]. Therefore, an efficient and eco-friendly strategy to improve plant adaptation can be an effective alternative method to improve plant yield under stressful conditions in sustainable agriculture [8,9].

The protective role of glycine betaine (GB) as an osmoprotectant in improving the adaptation of plants under abiotic stress has already been proven [10,11,12]. Although GB is naturally synthesized in some plants, GB synthesis is one of the most energy-intensive processes [13]. Therefore, the external use of GB in salinity-stressed plants can be an efficient way to improve plant adaptation. It has been shown that the use of GB improves the tolerance of plants under environmental stress by regulating the water potential and amplifying the antioxidant defense system [14,15]. GB also preserves the function of proteins by maintaining thermodynamic stability and reclaiming the aggregation of proteins, which diminish the peroxidation of membranes and, as a result, maintain the stability of cell membranes under salt stress [16]. It has been shown that transgenic plants containing GB synthesizing enzymes have high adaptability to environmental stresses such as salinity, drought, and low temperature, which indicates the role of GB as an important defensive compound under stressful conditions [17,18]. In several studies, the regulatory effects of GB on nitrogen and polyamine metabolism under environmental stress have been reported [12,19], which has attracted increasing attention in this field. However, the role of GB in regulating the plant’s vital metabolism, including the metabolism of polyamines and nitrogen under salt stress, has not been well studied.

*Stevia rebaudiana* Bertoni is one of the most important medicinal plants from the Asteraceae family, which is known as a sweet herb or sweet leaf due to its natural sweetener compounds (30 times sweeter than sugarcane) with zero calories [20]. Stevioside and rebaudioside (Reb A, B, C, etc.) are the main components of stevia sweeteners, which are used as natural sweeteners in the pharmaceutical, food, and beverage industries today. Stevia plant glycosides have been shown to provide a number of therapeutic benefits, including improved insulin sensitivity, the prevention of arteriosclerosis, the regulation of blood pressure, the lowering of blood sugar levels in diabetics, and the prevention of certain malignancies [21]. Considering the relative sensitivity of the stevia plant to salinity, the growth and secondary metabolites of the plant can be affected by salinity toxicity [22]. GB, as a natural substance synthesized in plants, has great potential for adjusting the adaptation of medicinal plants under stressful conditions, including salinity [12]. Despite the existing reports of the beneficial impacts of GB application in the adaptation of different plants under salt stress, there are few studies on the function of GB in controlling the metabolism of polyamines and the synthesis of secondary metabolites of the medicinal plant stevia, which has limited its use in stevia plants. Therefore, the interaction effects of NaCl and GB in in vitro conditions on growth, the antioxidant defense system, nitrogen and polyamine metabolism, as well as the expression of stevia glycoside synthesis enzymes, were investigated. The results obtained here are expected to improve our knowledge of GB-induced defense mechanisms under NaCl toxicity in stevia.

## 2. Materials and Methods

### 2.1. Preparing Samples and Applying Treatments

Stevia explants (non-lignified stems containing one node) were surface-disinfected with NaOCl for 10 min, and after washing with autoclaved distilled water, they were transferred to the modified MS medium [23]. The MS media were supplemented with 30 g L^−1^ sucrose, 1/2-strength microelements, 250 mg L^−1^ casein hydrolysate, and 8 g L^−1^ agar at pH 5.8. The samples were kept at a temperature of 25 ± 2 °C with 14 h of light and a light intensity of 100 μmol m^−2^ s^−1^ for 4 weeks. After 4 weeks, stevia shoot tips were cultured in jam jars with 50 mL of MS medium (as described above) supplemented with NaCl (0 and 50 mM) and GB (0, 25, and 50 mM). The treatments used are as follows: (a) control (no NaCl and GB), (b) 25 mM GB, (c) 50 mM NaCl, (d) 50 mM NaCl, (e) 50 mM NaCl + 25 mM GB, and (f) 50 mM NaCl + 50 mM GB. After 4 weeks, sampling was done to analyze biochemical and molecular traits.

### 2.2. Growth Parameters and Photosynthetic Pigments

After counting the number of roots and nodes, the samples were incubated for 48 h at 72 °C, and the total dry weight was obtained. To measure carotenoids and chlorophyll a and b, fresh leaf tissue was homogenized with 80% acetone, and after centrifugation, the supernatants were used to measure photosynthetic pigments as per Arnon [24].

### 2.3. Free Polyamines

After homogenizing the fresh leaves with cold perchloric acid (5% *v*/*v*) and incubating them on ice for 1 h, the supernatant solutions were mixed with benzoyl chloride and 2 N sodium hydroxide. After centrifugation, diethyl ether and saturated sodium chloride were added to the supernatant solutions. After centrifuging the samples and evaporating the diethyl ether phase, the remaining sediments were re-dissolved with methanol. Following, free polyamines (spermine (Spm), spermidine (Spd), and putrescine (Put)) were analyzed using an HPLC (Waters, Milford, MA, USA) and quantified through spectrofluorometrics with emission and excitation wavelengths of 510 and 365 nm, respectively, as per Naka et al. [25].

### 2.4. Methionine and Arginine

The protocol of Noctor and Foyer [26] was used to quantify methionine and arginine leaf contents through derivatization with o-phthalaldehyde. After homogenization of fresh leaves with HCl (0.1 M) and centrifugation, the supernatant solution was mixed with o-phthalaldehyde and measured using an HPLC device with a fluorescence detector (RF-20AXS, Tokyo, Japan). The guard column (15 × 3.2 mm, Shim-Pack, Tokyo, Japan) placed on the ODS Spheri 5 column (5 µm, 4.6 × 250 mm, GL Science Inc., Torrance, CA, USA) was used to separate the compounds.

### 2.5. Hydrogen Peroxide and Malondialdehyde

After extracting the leaves with trichloroacetic acid (1%) and adding potassium iodide (1 M) and potassium phosphate buffer (10 mM, pH 7.0) to the supernatants, the hydrogen peroxide (H_2_O_2_) content of the leaves was obtained by reading the mixture solution at 390 nm as per Loreto and Velikova [27].

To measure the content of malondialdehyde (MDA), after extracting fresh leaves with trichloroacetic acid (1%), the supernatant solutions were mixed with thiobarbituric acid (0.5%) in trichloroacetic acid (20%). Then, by placing the reaction mixture at 96 °C for 25 min, the absorbance of cooled samples was recorded at 532 nm, and the content of MDA was determined as per Hodges et al. [28].

### 2.6. Relative Water Content and Membrane Stability Index

After preparing the leaf discs and weighing them (W1), the leaf pieces were placed in distilled water at 25 °C for 4 h, and then their turgid weight was recorded (W2). Subsequently, leaf pieces were dried at 78 °C for 24 h and weighed (W3). The relative water content (RWC) was obtained as per the equation previously reported by Turner and Kramer [29]:

RWC = [(W1 − W3)/(W2 − W3)] × 100(1)

After incubating the leaf discs placed in distilled water at 25 °C and recording the electrical conductivity (EC1), the samples were autoclaved at 120 °C for 20 min. After obtaining EC2, the membrane stability index (MSI) was calculated as MSI (%) = [1 − {C1/C2}] × 100.

### 2.7. Activity of Polyamine Catabolizing Enzymes

Enzyme extract was prepared from fresh leaves using an extraction buffer containing potassium phosphate buffer (100 mM, pH 6.5) and dithiothreitol (5 mM). The method of Asthir et al. [30] was used to measure the activity of polyamine oxidase (PAO) and diamine oxidase (DAO) enzymes. By reading the absorbance of reaction mixtures containing enzyme extract, o-aminobenzaldehyde (1%), catalase (CAT, 50 U), potassium phosphate buffer (50 mM, pH 6.0), and Spd (10 mM) for PAO enzyme and enzyme extract, o-aminobenzaldehyde (1%), CAT (50 U) potassium phosphate buffer (50 mM, pH 7.5), and Put (10 mM) for DAO enzyme at 430 nm, leaf activity of polyamine catabolizing enzymes was obtained.

### 2.8. Activity of Antioxidant Enzymes

After the homogenization of fresh leaves with extraction buffer (50 mM TRIS buffer (pH 7.8), polyvinylpyrrolidone (7.5%), and EDTA-Na (1 mM)) and centrifugation, the supernatant solutions were used as enzyme extracts to determine the activities of superoxide dismutase (SOD), peroxidase (POD), catalase (CAT), and glutathione reductase (GR) enzymes. The activity of SOD and POD enzymes in stevia plant leaves was measured according to the methods of Nakano and Asada [31] and Cakmak and Marschner [32], respectively. The methods reported by Foyer and Halliwell [33] and Zhou and Leul [34] were used to measure the activities of GR and CAT enzymes, respectively.

### 2.9. Nitrogen and Nitrate Content

Dried leaf tissue was used to measure nitrogen content according to the micro-Kjeldahl method. To measure nitrate, after digesting dry leaf tissue in acetic acid (2%), a mixed solution containing N-1-naphthylethylenediamine dihydrochloride, sulfanilamide, powdered zinc, manganese sulfate monohydrate, and citric acid was added to the samples. After recording the absorbance of the samples at 540 nm, the nitrate content was quantified as per Singh [35].

### 2.10. Activity of Nitrogen Metabolism Enzymes

Extraction of fresh leaves of the stevia plant was done using extraction buffer (potassium phosphate buffer (100 mM, pH 7.5), cysteine (5 mM), PVP (0.5%), and EDTA (2 mM)), and supernatant solutions were used to measure nitrogen metabolism enzymes. The method of Debouba et al. [36] was used to measure the activity of nitrate reductase (NR) by reading the reaction mixture containing potassium phosphate buffer (100 mM, pH 7.5), KNO_3_ (7 mM), NADH (0.14 mM), the enzyme extract, zinc acetate (0.5 M), and MgCl_2_ (10 mM) at 540 nm, and the activity of nitrite reductase (NiR) by reading the reaction solution containing potassium phosphate buffer (0.1 M, pH 6.8), the enzyme extract, sodium dithionite (4.3 mM), NaNO_2_ (0.4 mM), and methyl viologen (2.3 mM) at 540 nm in stevia leaves.

The reaction mixture, including enzyme extract, Tris–HCl buffer (50 mM, pH 7.2), sodium arsenate (20 mM), glutamine (50 mM), hydroxylamine (13 mM), ADP (1 mM), HCl (0.5 M), MgCl_2_ (20 mM), and FeCl_3_ (0.2 M), was used to measure the leaf activity of glutamine synthetase (GS) as per Agbaria et al. [37] by recording the absorbance at 540 nm. The protocol of Groat and Vance [38] was used to measure glutamate synthase (GOGAT) activity by reading the absorbance of the reaction mixture (potassium phosphate buffer (50 mM, pH 7.5), phenylmethylsulfonyl fluoride (1 mM), EDTA (2 mM), KCl (10 mM), ethylene glycol (3.58 M), and β-mercaptoethanol (14 mM)) at 340 nm.

### 2.11. Sodium and Potassium Concentration

The concentrations of sodium (Na^+^) and potassium (K^+^) in stevia leaves were obtained using flame photometry (PFP7; Jenway, UK).

### 2.12. Diterpene Glycosides

To quantify the leaf content of stevioside and Reb A as previously reported by Yang et al. [39], dried stevia leaves were extracted with distilled water for 3 h at 80 °C. After adding the calcium chloride:ferrous sulfate (5:3) solution and centrifugation, the supernatant solutions were diluted with double-autoclaved distilled water. After filtering the solution with a PTFE filter (0.45 μm pore size), the samples were analyzed using HPLC (Shimadzu, Deurne, Belgium) as previously described by Ghasemi-Omran et al. [22].

### 2.13. Gene Expression

Total mRNA extraction and cDNA synthesis were performed using TRIzol (Invitrogen, Carlsbad, CA, USA) and reverse transcriptase kits (Thermo Fisher Scientific, Waltham, MA, USA), respectively, as per the company’s instructions. SYBR Green Master Mix (2X, Thermo Scientific, Waltham, MA, USA) was used to perform qPCR using the C1000^TM^ Thermal Cycler (BioRad, Hercules, CA, USA) and the *Actin* gene for normalization. The relative expression of the target genes in the leaves of the stevia plant was calculated based on the 2^−ΔΔCT^ method with three independent biological replicates. The primer sequences of the target genes are given in Table S1.

### 2.14. Statistical Analyses

The SAS (V9, SAS Institute Inc., Cary, NC, USA) program was used to analyze the data. The mean (± SD, n = 6) comparison was done by the Duncan test at a 5% probability level [40].

## 3. Results

### 3.1. Growth and Photosynthesis Traits

The results showed that adding 50 mM NaCl to the MS medium diminished the number of nodes and the number of roots compared to the control plants by 54.8 and 35.4%, respectively. In the control plants, adding GB at concentrations of 25 and 50 mM did not induce a significant effect on the number of nodes, but the 25 mM treatment caused a significant increase in the number of roots by 7% compared to the control plants. In NCl-stressed plants, the application of both concentrations of GB caused a significant increase in the number of nodes and the number of roots (with the highest observed at 50 mM) compared to NaCl treatments alone (Table 1). A significant decrease in total dry weight was observed in NaCl and NaCl + GB (25 and 50 mM) treatments compared to control plants, and the largest decrease was recorded in NaCl treatments (Table 1).

NaCl treatment decreased the contents of chlorophyll a (46.6%), chlorophyll b (41.8%), and carotenoids (40.5%) compared to plants grown in a NaCl-free MS medium. However, adding GB to the MS culture medium containing NaCl improved the photosynthetic pigments (Table 1).

### 3.2. Metabolism of Polyamines

**Figure 1:**
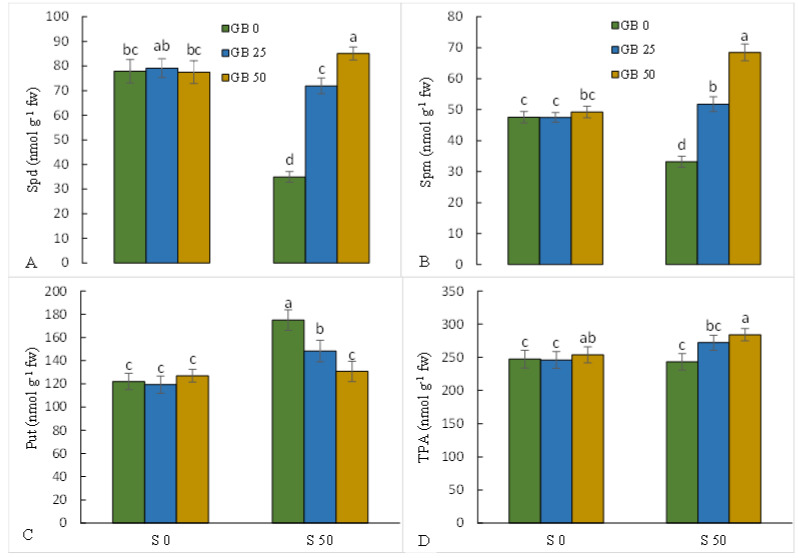
Effect of glycine betaine (GB, 0 and 50 mM) on the leaf contents of spermidine (Spd, (**A**)), spermine (Spm, (**B**)), putrescine (Put, (**C**)), and total polyamine (TPA, (**D**)) in the stevia plant under NaCl (0, 25, and 50 mM) treatment. Values (means ± SD, n = 6) followed by the same letter are not significantly different (*p* < 0.05; Duncan test).

Analysis of polyamines showed that NaCl treatment decreased Spd (55.2%) and Spm (30.2%) and increased Put (43.4%) in stevia leaves compared to plants without NaCl treatment. However, the addition of GB to NaCl-stressed plants increased Spd and Spm and decreased Put (Figure 1A–C). NaCl treatment did not induce a significant effect on total polyamine. However, GB increased total polyamines in NaCl-stressed plants. (Figure 1D).

**Figure 2:**
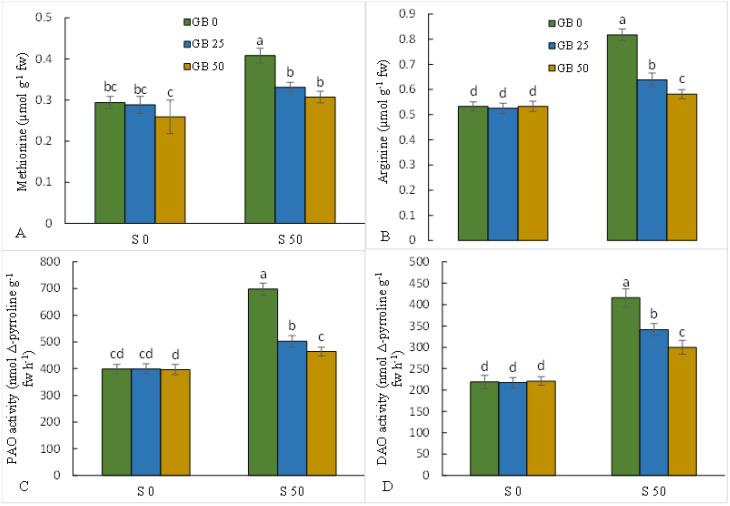
Effect of glycine betaine (GB, 0 and 50 mM) on the content of methionine (**A**) and arginine (**B**) and the activities of polyamine oxidase (PAO, (**C**)) and diamine oxidase (DAO, (**D**)) of the stevia leaves under NaCl (0, 25, and 50 mM) treatment. Values (means ± SD, n = 6) followed by the same letter are not significantly different (*p* < 0.05; Duncan test).

An increase of 28 and 53.6% was recorded in the leaf content of methionine and arginine, respectively, under NaCl stress compared to plants without NaCl. However, the application of GB simultaneously with salinity treatment decreased leaf levels of each amino acid, methionine, and arginine (Figure 2A,B). The activity of PAO and DAO enzymes increased by 75.4 and 90%, respectively, by adding NaCl to the MS medium. However, the use of GB simultaneously with NaCl significantly decreased the activity of both enzymes in stevia leaves (with the highest decrease recorded under 50 mM GB) (Figure 2C,D).

### 3.3. Oxidative Stress

**Figure 3:**
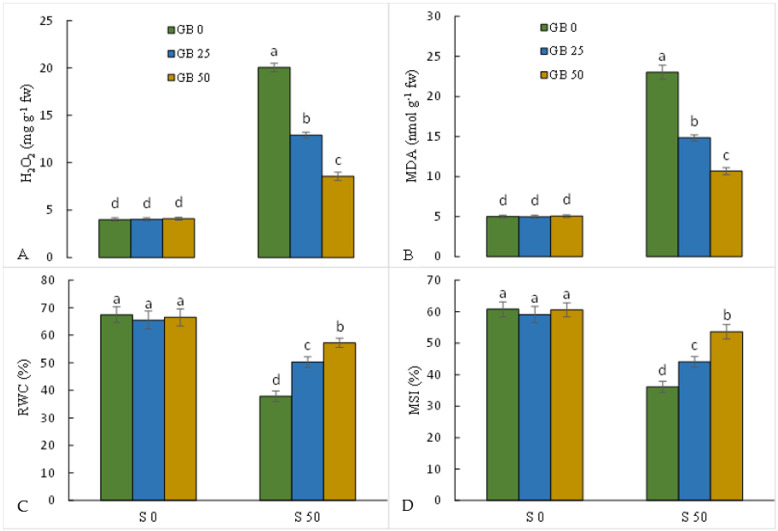
Effect of glycine betaine (GB, 0 and 50 mM) on the content of hydrogen peroxide (H_2_O_2_, (**A**)) and malondialdehyde (MDA, (**B**)), relative water content (RWC, (**C**)), and membrane stability index (MSI, (**D**)) of stevia leaves under NaCl (0, 25, and 50 mM) treatment. Values (means ± SD, n = 6) followed by the same letter are not significantly different (*p* < 0.05; Duncan test).

According to the obtained results, the addition of 50 mM NaCl caused an increase in H_2_O_2_ and MDA of 5 and 4.6 fold, respectively, and a decrease in RWC and MSI of 43.9 and 40.6%, respectively, in leaves compared to plants grown in MS without NaCl. However, the simultaneous application of GB with NaCl in a concentration-dependent manner caused a decrease in the leaf level of H_2_O_2_ and MDA and an increase in RWC and MSI in stevia leaves compared to plants grown on NaCl alone containing MS (Figure 3A–D).

**Figure 4:**
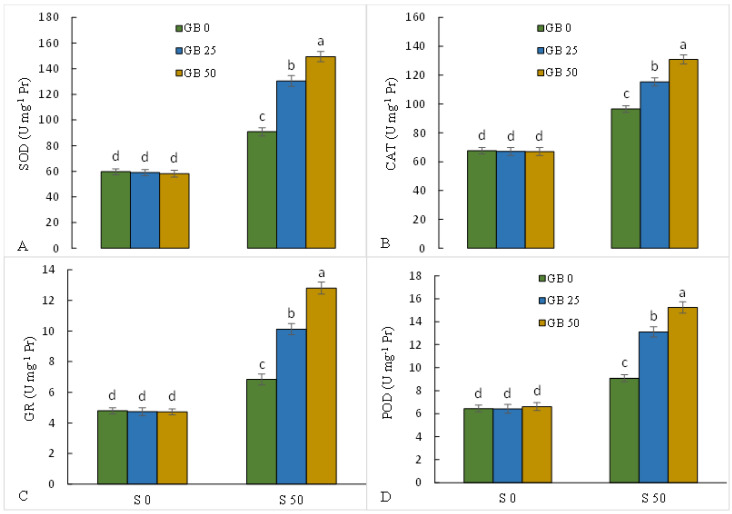
Effect of glycine betaine (GB, 0, and 50 mM) on the activity of antioxidant enzymes (superoxide dismutase (SOD, (**A**)), catalase (CAT, (**B**)), glutathione reductase (GR, (**C**)), and peroxidase (POD, (**D**))) in stevia leaves under NaCl (0, 25, and 50 mM) treatment. Values (means ± SD, n = 6) followed by the same letter are not significantly different (*p* < 0.05; Duncan test).

When 50 mM NaCl was added to the MS medium, the activities of SOD, CAT, GR, and POD enzymes were raised by 52.2, 42.8, 42.8, and 40.8%, respectively, in stevia leaves compared to plants without NaCl. However, with the addition of GB to the culture medium containing NaCl, the activity of the above-mentioned antioxidant enzymes showed a greater increase, and the highest increase effect was found under the treatment of 50 mM GB (Figure 4A–D).

### 3.4. K/Na Homeostasis

NaCl stress caused a decrease in K accumulation and an increase in Na accumulation in leaves, which was accompanied by a decrease in the K/Na ratio. However, the simultaneous application of GB and NaCl increased K accumulation and the K/Na ratio and decreased Na (Table 2).

### 3.5. Nitrogen Metabolism

In control plants, the application of NaCl stress lowered nitrogen concentration by 46% and nitrate concentration by 62.5% in stevia leaves. The GB treatment did not cause significant changes in nitrogen and nitrate concentrations in control plants; however, it increased nitrogen and nitrate concentrations in NaCl-stressed stevia leaves (Table 2).

**Figure 5:**
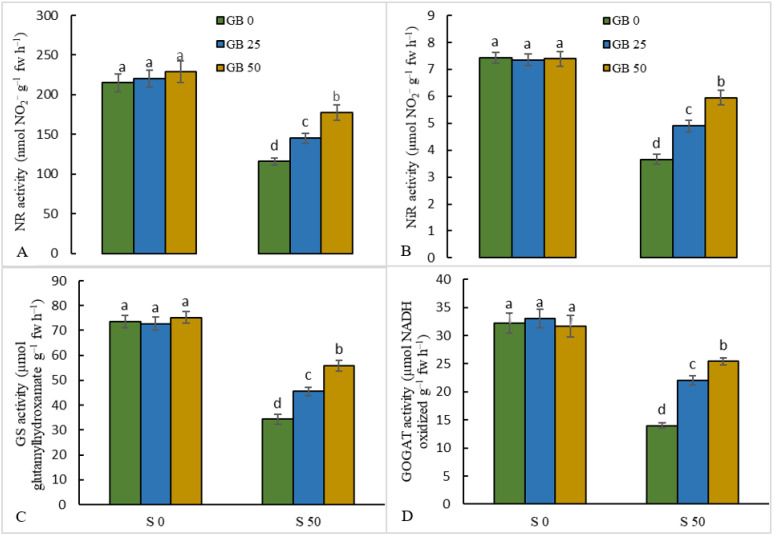
Effect of glycine betaine (GB, 0, and 50 mM) on the activity of N-metabolism enzymes (nitrate reductase (NR, (**A**)), nitrite reductase (NiR, (**B**)), glutamine synthetase (GS, (**C**)), glutamate synthase (GOGAT, (**D**))) in stevia leaves under NaCl (0, 25, and 50 mM) treatment. Values (means ± SD, n = 6) followed by the same letter are not significantly different (*p* < 0.05; Duncan test).

The results related to the activity of enzymes involved in nitrogen metabolism showed that when 50 mM NaCl was added to the MS culture medium of the stevia plant, the activities of NR (46.1%), NiR (50.8%), GS (53.3%), and GOGAT (56.6%) enzymes were significantly decreased in comparison to control samples. However, in NaCl-stressed plants, treatments with 25 and 50 mM GB upregulated the activities of NR by 25.3 and 53.5%, NiR by 33.9 and 62.6%, GS by 32.7 and 62.6%, and GOGAT by 57.6 and 81.8%, respectively, compared to plants treated with NaCl alone (Figure 5A–D).

### 3.6. Diterpene Glycosides

**Figure 6:**
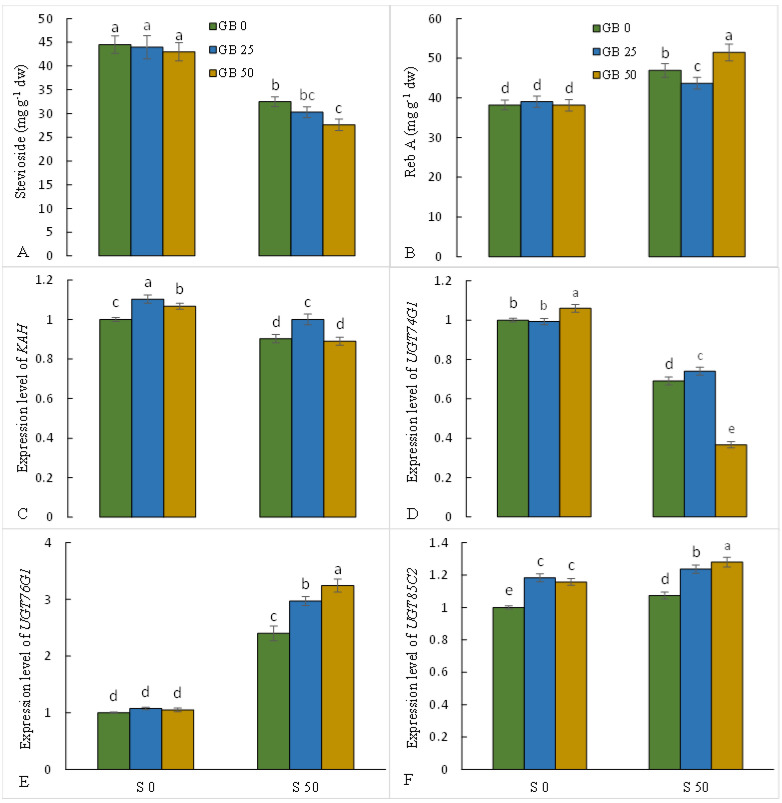
Effect of glycine betaine (GB, 0 and 50 mM) on the content of stevioside (**A**) and rebaudioside (Reb) A (**B**) and the expression level of the *KAH* (**C**), *UGT74G1* (**D**), *UGT76G1* (**E**), and *UGT85C2* (**F**) genes of stevia leaves under NaCl (0, 25, and 50 mM) treatment. Values (means ± SD) followed by the same letter are not significantly different (*p* < 0.05; Duncan test).

The treatment with 50 mM NaCl provoked a 27% decline in the accumulation of stevioside and a 22.8% increase in the accumulation of Reb A compared to the control plants. No significant difference was observed in the leaf accumulation of stevioside and Reb A under GB treatments in plants without NaCl treatment. However, GB treatments in NaCl-exposed plants resulted in a further decrease in stevioside levels in leaves. Different concentrations of GB induced different responses in the level of Reb A in NaCl-stressed plants. 25 mM GB decreased Reb A by 6.9%, and 50 mM glycine treatment increased Reb A by 9.7% in NaCl-stressed plants (Figure 6A,B).

Analyzing the expression of genes synthesizing diterpene glycosides in stevia showed that the expression of *KAH* and *UGT74G1* genes in the leaves of NaCl-stressed plants decreased; however, the expression of *UGT76G1* and *UGT85C2* genes in the leaves of NaCl-stressed plants was associated with a significant enhancement compared to control plants. In control plants, 25 and 50 mM GB treatments increased the expression of *KAH* and *UGT85C2* genes, and 50 mM GB treatment significantly increased *UGT74G1* gene expression, while GB treatments did not induce significant differences in *UGT76G1* gene expression. In NaCl-stressed plants, 25 mM GB treatment enhanced the expression of *KAH* and *UGT74G1*, and 25 and 50 mM GB treatments upregulated *UGT76G1* and *UGT85C2* gene expression in stevia leaves, while 50 mM GB treatment decreased *UGT74G1* expression compared to plants treated with salt alone (Figure 6C–F).

## 4. Discussion

It is well accepted that the high concentration of NaCl in the rhizosphere can reduce the growth and yield of plants; however, the amount of damage to plants is greatly influenced by the concentration and duration of exposure to stress and the plant species. Although a lot of research has been done in order to know the responses of model plants and important crops under NaCl toxicity, investigating the effect of salinity on the biosynthetic pathway of secondary metabolites as well as the defense mechanisms of medicinal plants seems necessary. In addition, tissue culture has provided a dynamic and powerful approach to investigating the response of plants to abiotic stresses, including NaCl stress, under controlled conditions [41]. The results revealed that applying 50 mM NaCl significantly lowered chlorophyll *a*, *b*, and carotenoids in the stevia plant, which indicates damage to the photosynthetic apparatus and was accompanied by a decrease in the number of nodes, number of roots, and total dry weight. Similarly, the reduction of photosynthetic pigments and growth in the stevia plant due to NaCl toxicity has been previously reported by Shahverdi et al. [42], Lucho et al. [41], and Ghasemi-Omran et al. [22]. The NaCl-induced adverse effects on growth and photosynthetic pigments can be provoked by the induction of oxidative stress [43], disruption of ionic homeostasis [44], and high concentrations of Na and Cl [45], which damage the protein complex of reaction centers in thylakoid membranes and disturb the biosynthetic pathway of chlorophylls [46]. However, the application of GB significantly improved the growth and photosynthetic pigments in NaCl-stressed stevia plants, which indicates the beneficial role of GB in the adaptation of stevia plants to NaCl toxicity. The positive role of the external application of GB on chlorophyll biosynthesis under NaCl stress in wheat [47], canola [48], and rice [49] plants has been previously documented. Sofy et al. [50] displayed that GB reduces the level of toxic radicals caused by salt stress by strengthening the antioxidant defense system, which maintains the photosynthetic pigments by protecting the thylakoid membranes and the protein complex of the photosynthetic apparatus. It has been stated that the protective effect of GB on photosynthetic pigments in NaCl-stressed plants can be caused by increasing the absorption of Mg as an essential element in chlorophyll biosynthesis, alleviating the accumulation of toxic Na, raising the internal level of cytokinin, delaying the degradation of chlorophyll, lowering the activity of the chlorophyllase enzyme, and restoring K/Na homeostasis [51,52,53].

NaCl stress, by increasing the accumulation of Na and Cl and reducing the absorption of nutrients, disrupts ionic homeostasis, especially the ratio of K/Na in plants, which induces serious damage to the vital processes and metabolisms of the plant. Therefore, maintaining K/Na homeostasis can play an important role in plant adaptation to NaCl toxicity. Our results showed that NaCl stress raised leaf Na accumulation and decreased leaf K accumulation and the K/Na ratio, which can indicate damage to membrane lipids and increased ion leakage. Similar results of disruption of K/Na homeostasis in stevia plants [22] and other plant species [12,44] have been reported previously. It has been stated that disruption of K/Na homeostasis in NaCl-stressed plants can be caused by the induction of oxidative stress and disturbance in the uptake mechanism of nutrients in the roots [45,50]. However, the application of GB in the culture medium of NaCl-stressed plants caused a decrease in Na leaf accumulation and an increase in K leaf accumulation and, as a result, the restoration of the K/Na ratio, which was supported by the results of Sofy et al. [50] and Zhu et al. [12]. Mahmood et al. [54] showed that GB reduces Na translocation to leaves by transferring Na into the vacuoles of root cells. The application of GB has been shown to effectively diminish K efflux in NaCl-stressed plants [55]. Zhu et al. [12] showed that GB decreased Na uptake in roots and increased Na efflux in leaf cells by upregulating vacuolar NHX, PM Na^+^ transporters, and PM H^+^-ATPase expression and inducing PM H^+^-ATPase activity. However, the exact role of GB in maintaining K/Na homeostasis is not well known, which requires more research at the molecular level, focusing on the transporters involved in the uptake and transport of K and Na under NaCl stress.

NaCl stress, by disrupting the main processes of plant cells, including respiration, photosynthesis, amine oxidases, and NADPH oxidases, increases the production of ROS and damages the vital metabolism and processes in plants [56]. NaCl-stressed plants were associated with an increase in the level of H_2_O_2_ and MDA and a decrease in RWC and MSI compared to control plants, which indicates the induction of oxidative stress and damage to the integrity of biomembranes. The damage caused by high levels of toxic radicals and ROS induced by salinity to thylakoid membranes and protein complexes in the reaction centers can intensify the oxidative stress in the plant [57]. Therefore, strengthening the antioxidant defense system to reduce the level of ROS and protect bio-macromolecules can play a critical role in improving adaptation in NaCl-stressed plants. Our results showed that the application of GB significantly upregulated the leaf activity of antioxidant enzymes (CAT, GR, POD, and SOD) in NaCl-stressed plants, which corresponds to the reduction of the level of toxic ROS and MDA. In fact, GB diminished NaCl stress-induced oxidative stress in the stevia plant by inducing the antioxidant defense system, which can significantly enhance the adaptation of the stevia plant under NaCl toxicity. It has been shown that increasing the internal level of GB in transgenic plants not only preserves the activity of antioxidant enzymes but also stabilizes the structure of proteins, including enzymes involved in amino acid and sugar metabolism under environmental stress [17,58]. It has also been shown that by binding to membrane phospholipids, GB can reduce oxidative stress-induced damage to lipid membranes [18,59]. The induction effect of GB on antioxidant enzymes and reduction of NaCl-induced oxidative stress was supported by the results previously reported by Hasanuzzaman et al. [49], Desoky et al. [60], and Sofy et al. [50].

Salinity stress causes irreparable damage to plant growth and development by damaging the main metabolisms, including the N metabolism. Therefore, maintaining N metabolism can play a potential role in increasing the plant’s ability to deal with the adverse effects of NaCl toxicity [61,62]. The results revealed that the application of NaCl treatment decreased the concentration of N and NO_3_ and downregulated the activity of NR, NiR, GS, and GOGAT enzymes in stevia leaves, which indicates a disturbance in N absorption and metabolism. Similar results of the negative effects of salinity stress on N metabolism have been documented by Talaat [63] and Hussain et al. [64]. The increase of NaCl-induced toxic ROS and damage to membrane integrity can be one of the reasons for reduced N uptake and damage to its assimilation [63]. An excessive increase in ROS and toxic radicals can also cause damage to the structure of proteins, including enzymes of the N metabolism pathway, and, as a result, disrupt their function [65]. In NaCl-stressed plants, the application of GB increased the activity of N metabolism enzymes and the leaf accumulation of N and NO_3_, which indicates the positive role of GB on N assimilation under NaCl toxicity. In other reports, it was shown that the induction of N metabolism enzymes improves plant adaptation to environmental stresses, which indicates the importance of maintaining N metabolism to strengthen plant adaptation under stressful conditions, especially NaCl toxicity [3]. Given the role of GB as an active osmolyte and in inducing the antioxidant defense system [66], the positive effect of GB on N metabolism can be caused by diminishing oxidative stress and protecting the structure of N metabolism enzymes from NaCl toxicity. By regulating the water potential of cells and improving the relative water content [14,59], GB can improve the function of enzymes involved in N metabolism and restore N assimilation under water stress induced by high NaCl concentration. However, more detailed studies at the molecular level are needed to understand the regulatory role of GB on N metabolism under NaCl toxicity.

Polyamines play a critical role in plant growth and development by regulating cell division in fruit ripening, floral development, senescence, embryogenesis, and rhizogenesis. It has been proven that polyamines, as signaling molecules, play an effective role in plant response to environmental stresses [3,67]. NaCl treatment decreased the leaf activity of PAO (which degrades Spd and Spm) and DAO (which degrades Put) enzymes, which is consistent with the reduction of Spd, Spm, and Put content in leaves. In fact, NaCl toxicity, by inducing enzymes that decompose polyamines, caused a decrease in the leaf content of polyamines in the stevia plant, which is supported by the results obtained by Talaat [63] and Ke et al. [3]. ElSayed et al. [68] showed that salinity stress by upregulating the expression of *PAO* and *DAO* increased the activity of these enzymes in rapeseed plants. Considering the high requirement of N for the synthesis of polyamines [69], the reduction of uptake and assimilation of N under NaCl stress can be another factor contributing to the diminished content of polyamines in NaCl-stressed stevia. By reducing the activity of enzymes involved in the degradation of polyamines, GB increased the leaf content of polyamines in NaCl-stressed plants, which indicates the role of GB in modulating the metabolism of polyamines under NaCl toxicity. Considering the positive role of polyamines in improving plant tolerance to salinity stress [70], the GB-induced increase in the leaf content of polyamines can play an important role in improving the adaptability of stevia plants during NaCl toxicity. Similarly, Liu et al. [71] showed that the exogenous application of GB improved plant adaptation under salinity stress by increasing the endogenous content of polyamines. The positive effects of GB on the metabolism of polyamines could be due to the availability of more N in GB-treated stevia plants under NaCl stress, as previously supported by Talaat [63] and Ke et al. [3]. Therefore, our results revealed that GB, by modulating the metabolism of polyamines, increased the leaf accumulation of these important defensive compounds under NaCl toxicity, which can effectively improve the adaptation of NaCl-stressed stevia plants.

Steviol glycosides in stevia plants include a group of valuable secondary metabolites that are biosynthesized from mono-, di-, and tetra-terpene pathways; however, the role of these compounds during environmental stress, including salinity stress, is not well known [21]. The results showed that NaCl treatment decreased the expression of *KAH* and *UGT74G1* genes and increased the expression of *UGT76G1* and *UGT85C2* genes in stevia leaves, which was associated with a decline in the leaf content of stevioside and an increase in the leaf content of Reb A. The results suggest that NaCl stress modulated the conversion of stevioside to Reb A by inducing *UGT76G1* gene expression. Similarly, Zeng et al. [72] indicated that salt stress increased the leaf accumulation of Reb A in the Stevia plant, which was caused by the up-regulation of *UGT76G1* gene expression. Ceunen and Geuns [21] demonstrated that steviol glycosides, especially Reb A, play a critical role as osmo-protectant metabolites in osmotic regulation under water stress. Therefore, the increase in the accumulation of Reb A could indicate the induction of a defense mechanism to cope with salinity-induced water stress. The results revealed that GB increased the accumulation of Reb A by modulating the expression of genes biosynthesizing secondary metabolites of stevia, which can indicate the defensive role of this compound in dealing with NaCl toxicity; however, more research is required to confirm these concepts.

## 5. Conclusions

According to the obtained results, it can be concluded that the external application of GB improved the growth and adaptation of stevia plants under NaCl toxicity through the adjustment of N and PAO metabolisms, the improvement of the antioxidant defense system, and the maintenance of K/Na balance. GB treatment also increased the accumulation of Reb A in the leaves of stevia by modulating the expression of enzymes synthesizing steviol glycosides, which can indicate the defensive role of this compound during NaCl toxicity. Although the results of improving adaptation to NaCl stress induced by GB in other plants should be investigated, GB can be considered a promising and eco-friendly agent to enhance the growth and production of agricultural and horticultural crops under abiotic stresses.

## Figures and Tables

**Table 1:** Effect of glycine betaine (GB, 0 and 50 mM) on nodule number, root number, total dry weight, and photosynthetic pigments of the stevia plant under NaCl (0, 25, and 50 mM) treatment.

Treatments (mM)	N Node	N Root	TDW	Chl *a*	Chl *b*	Car
				mg/gFW		
Control	8.10 ± 0.26 ^a^	7.55 ± 0.20 ^b^	0.251 ± 0.022 ^a^	1.91 ± 0.15 ^a^	1.10 ± 0.08 ^a^	0.348 ± 0.023 ^a^
GB 25	8.39 ± 0.17 ^a^	8.08 ± 0.24 ^a^	0.275 ± 0.021 ^a^	2.03 ± 0.16 ^a^	1.08 ± 0.07 ^a^	0.354 ± 0.015 ^a^
GB 50	8.24 ± 0.13 ^a^	7.45 ± 0.21 ^bc^	0.251 ± 0.016 ^a^	2.05 ± 0.10 ^a^	1.04 ± 0.10 ^ab^	0.342 ± 0.024 ^a^
NaCl 50	3.66 ± 0.18 ^d^	4.88 ± 0.22 ^e^	0.121 ± 0.009 ^d^	1.02 ± 0.07 ^d^	0.64 ± 0.03 ^c^	0.207 ± 0.018 ^d^
NaCl 50 + GB 25	5.29 ± 0.15 ^c^	6.73 ± 0.23 ^d^	0.179 ± 0.011 ^c^	1.47 ± 0.08 ^c^	0.92 ± 0.04 ^b^	0.263 ± 0.016 ^c^
NaCl 50 + GB 50	6.24 ± 0.20 ^b^	7.10 ± 0.23 ^cd^	0.209 ± 0.009 ^b^	1.69 ± 0.09 ^b^	0.99 ± 0.04 ^ab^	0.299 ± 0.016 ^b^

Values (means ± SD, n = 6) followed by the same letter are not significantly different (*p* < 0.05; Duncan test).

**Table 2:** Effect of glycine betaine (GB, 0, and 50 mM) on the leaf concentrations of K and Na, the K/Na ratio, and the leaf concentrations of N and NO_3_ of the stevia plant under NaCl (0, 25, and 50 mM) treatment.

Treatments (mM)	K	Na	K/Na	N (mg/gDW)	NO_3_ (µmol/gfw)
	mg/gDW				
Control	4.81 ± 0.30 ^ab^	0.72 ± 0.04 ^d^	6.70 ± 0.16 ^a^	33.07 ± 0.60 ^a^	28.17 ± 0.72 ^a^
GB 25	4.81 ± 0.30 ^ab^	0.73 ± 0.03 ^d^	6.56 ± 0.12 ^a^	32.26 ± 0.55 ^a^	26.96 ± 1.20 ^a^
GB 50	4.90 ± 0.27 ^a^	0.72 ± 0.04 ^d^	6.82 ± 0.05 ^a^	32.44 ± 0.79 ^a^	27.39 ± 0.79 ^a^
NaCl 50	2.99 ± 0.15 ^d^	2.46 ± 0.10 ^a^	1.22 ± 0.02 ^d^	17.87 ± 0.60 ^d^	13.70 ± 0.44 ^d^
NaCl 50 + GB 25	3.96 ± 0.16 ^c^	1.30 ± 0.06 ^b^	3.06 ± 0.26 ^c^	25.33 ± 0.43 ^c^	20.91 ± 0.63 ^c^
NaCl 50 + GB 50	4.39 ± 0.14 ^b^	1.02 ± 0.10 ^c^	4.36 ± 0.53 ^b^	28.25 ± 0.43 ^b^	22.96 ± 0.64 ^b^

Values (means ± SD, n = 6) followed by the same letter are not significantly different (*p* < 0.05; Duncan test).

## Data Availability

Not applicable.

## Supplementary Materials

The following supporting information can be downloaded at: https://www.mdpi.com/article/10.3390/plants12081628/s1, Table S1. The primers sequences used in qPCR reactions.
