# Supplementary material for: The Effect of Glycine Betaine on Nitrogen and Polyamine Metabolisms, Expression of Glycoside-Related Biosynthetic Enzymes, and K/Na Balance of Stevia under Salt Stress
Source: Plants (Basel). 2023 Apr 12;12(8):1628. doi: 10.3390/plants12081628 (PMC10141388; doi:10.3390/plants12081628)
Supplement: Supplementary file 1 [file plants-12-01628-s001.zip › plants-2273620-supplementary.pdf]

Table S1. The primers sequences used in qPCR reactions.

Gene	Primer sequence 5′→3′ (forward/reverse)	Amplicon length (bp)	Accession No.
Actin	F: TCGAACACGGTATTGTCAGC	143	AF548026
	R: CTTTTCTCTGTTCGCCTTGG		
Kaurenoic acid hydroxylase (KAH)	F: AGCTTTTCGGCAAGTCTCTG	116	EU722415
	R: CATGGTGACGGCATAATGAG		
UGT74G1	F: TCCTGGATTTCCAGTGCTTC	80	AY345982
	R: GAGACCAAGGGCTCTGTATTTG		
UGT76G1	F: AATAGCTCGTGGGTTGGTTG	77	AY345974
	R: ACGTCGAACCCTTGACAAAC		
UGT85C2	F: CAAGAGTTGATGGGAGAAGGAG	137	AY345978
	R: AGCACGGTGATTTCCTTGAC		
